# Assessment of perinatal outcomes of pregnant women with severe versus simple malaria

**DOI:** 10.1371/journal.pone.0247053

**Published:** 2021-02-19

**Authors:** Alfred Kwizera, Diomede Ntasumumuyange, Maria Small, Stephen Rulisa, Alexandra N. Moscovitz, Urania Magriples

**Affiliations:** 1 Department of Obstetrics and Gynecology, College of Medicine and Health Sciences/University of Rwanda, Kigali, Rwanda; 2 University Teaching Hospital of Kigali, Kigali, Rwanda; 3 Department of Obstetrics and Gynecology, Yale University School of Medicine, New Haven, Connecticut, United States of America; 4 Department of Obstetrics and Gynecology, Duke University School of Medicine, Durham, North Carolina, United States of America; 5 Mailman School of Public Health, Columbia University, New York, New York, United States of America; Instituto Rene Rachou, BRAZIL

## Abstract

**Objective:**

Malaria in pregnancy is associated with adverse perinatal outcomes. The objective was to compare outcomes of simple and severe malaria and to determine whether they vary by trimester or severity of infection.

**Methods:**

Prospective cohort study performed in 3 hospitals in Rwanda. Both hospitalized and non-hospitalized pregnant patients with confirmed malaria were enrolled and followed until 7 days postpartum. Demographic, clinical manifestations and perinatal outcomes were recorded.

**Results:**

There were 446 pregnant women with confirmed malaria and outcome data; 361 (80.9%) had simple malaria. Severe malaria was more common as pregnancy progressed; out of 85 with severe malaria, 12.9%, 29.4% and 57.6% were in the 1^st^, 2^nd^ and 3^rd^ trimesters (p<0.0001). Overall, a normal term delivery occurred in 57.6%, with preterm delivery in 24.9% and abortion in 13.5%. Adverse perinatal outcomes increased with trimester of infection (p<0.0001). Eight of the 9 early neonatal deaths had 3^rd^ trimester infection (p<0.0001). There were 27 stillbirths; 63.7% were associated with 3^rd^ trimester infection. A significant difference in perinatal outcomes between simple and severe malaria was seen: 64% of women with simple malaria had a normal term delivery as compared to 30.6% with severe malaria (p<0.0001). All complications were significantly greater with severe malaria.

**Conclusion:**

Overall poor outcomes are seen in malaria with significant differences in perinatal outcomes between simple and severe malaria and by trimester of infection. In addition to vector control and exposure prevention, efforts need to be made in screening, treatment education and monitoring pregnancies affected by malaria.

## Introduction

Malaria remains a serious life threatening public health concern in sub-Saharan Africa with 25 million pregnant women affected by malaria, predominantly by Plasmodium falciparum [1]. Many studies have shown that in endemic areas, malaria in pregnancy is more common in young women, primigravidae and women infected with HIV [2, 3]. Multigravidas have developed a degree of immunity to the parasite from previous infections [3]. Women from hyperendemic areas with lifelong exposure to the malaria parasite have heightened immunity. In contrast, women in mesoendemic regions, such as in highland areas of Rwanda, have less exposure, and consequently less immunity, and therefore are at risk for more severe manifestations. Rwanda had made significant progress in malaria reduction from 2005 to 2012 with an 86% reduction in malaria incidence and a 74% reduction in mortality. However, between 2012 and 2016 there was an 8 fold increase with reported malaria cases reaching more than 2 million in 2015, leaving women of reproductive age particularly vulnerable as they are less likely to have immunity [1, 4, 5].

Rwanda’s malaria in pregnancy strategy includes distribution of insecticide-treated nets at the first antenatal care visit, iron and folate supplementation and prompt evaluation for malaria in pregnant women. Intermittent screening and treatment is not national policy but currently under investigation [6].

Severe disease is more common in pregnancy and maternal mortality is significantly increased in severe disease [7]. In endemic areas, malaria affects both maternal and neonatal outcomes. Maternal outcomes are complicated by anemia, pulmonary edema, cerebral edema, hypoglycemia and renal failure, and malaria has been associated with increased risk of abortion, low birth weight, preterm delivery, stillbirth and neonatal death [8–10]. Placental sequestration of infected erythrocytes is also hypothesized to contribute to morbidity and is associated with abortions, stillbirth, low birth weight, preterm deliveries, and increased neonatal mortality [11, 12]. Fever and anemia also contribute to poor perinatal outcomes. In sub-Saharan African countries including Rwanda, 75.000–200.000 infant deaths are attributed to malaria in pregnancy annually, and most of the deaths are caused indirectly by low birth weight and preterm deliveries [1, 8].

The purpose of this study was to identify adverse perinatal outcomes of pregnant women with malaria in Rwanda. Such comparison of perinatal outcomes remains relevant and significant for health care providers in deciding whether only patients with severe malaria, or all patients with malaria require more intensive fetal surveillance and monitoring for better perinatal outcomes in addition to anti-malarial drugs and routine antenatal consultations.

## Materials and methods

### Study design and setting

A prospective observational cohort study was conducted over a ten month period from May 2016 to February 2017 in three hospitals in Rwanda. Butare University Teaching Hospital (BUTH) is a tertiary referral center which has 2000 deliveries annually and is the referral hospital for 17 District Hospitals from the Southern and Western Provinces. Kibagabaga District Hospital (KDH) is a public hospital in Kigali with 5500 annual deliveries which provides care for low and moderate income pregnant women from endemic rural areas surrounding Kigali. Nyamata District Hospital (NDH) has 5000 annual deliveries and is located in Eastern Province. These three facilities were chosen as they represent a wide geographic distribution of patients in Rwanda and have high rates of malaria.

### Participants

Our study population included all pregnant women with malaria who were admitted or treated as outpatients during the study period at the three participating hospitals. Participants were enrolled in the inpatient or outpatient setting after confirmation of the diagnosis by malaria smear. Positive malaria smears were also tracked through the individual hospital laboratories to identify potential pregnant participants. Clinical management was not affected by study participation and no additional bloodwork outside of standard clinical practice was performed as part of the study. The exclusion criteria was refusal to participate in the study, hypertensive disorders, diabetes and twin gestation. A total of 484 pregnant women with severe and simple malaria were enrolled during the study period. No one refused participation. Follow up was unavailable for 38 patients (7.9%). Among the 446 women with complete follow up, 245 (54.9%) were from NDH, 109 (24.4%) from KDH and 91 (20.4%) were from BUTH.

### Variables and data source

Maternal demographic, prior obstetrical history and gestational age were recorded on enrollment. Gestational age was established by LMP or prior ultrasound if available. If there was not an established gestational age, an obstetric ultrasound for fetal biometry was performed with a Sonosite M-Turbo® portable during evaluation as part of routine obstetric care.

A clinical diagnosis of severe versus simple malaria was determined by WHO guidelines [13]. Clinical manifestations were recorded in order to categorize the cohort as simple or severe malaria. Simple malaria is defined as symptomatic malaria without laboratory or clinical evidence of severity or vital organ dysfunction. Symptoms include: Moderate fever 37.5°C or history of fever in the last 24 hours, headache, weakness, chills, loss of appetite, stiffness, joint pain, and muscular aches. If symptoms of simple malaria are associated with nausea, vomiting and diarrhea, they will therefore be defined as simple malaria with minor digestive disorders. Severe malaria is defined as malaria infection with clinical or laboratory evidence of any end organ dysfunction, manifested by impaired consciousness (Glasgow coma score<11), anemia (hemoglobin< 7g/dl), renal impairment, pulmonary edema, significant bleeding and shock. Serum creatinine was used for the evaluation of renal involvement as other measures of renal function such as glomerular filtration rate or electrolytes are not routinely obtained. Hemoglobin, platelet count, and mean red cell corpuscular volume were recorded on all patients to ascertain pregnant women admitted with anemia or thrombocytopenia associated with malaria. MCV was used to distinguish between anemia due to hemolysis (normocytic) or iron deficiency (microcytic). Glucose was evaluated by fingerstick on admission in all patients and hypoglycemia was defined as a value of less than 70 mg/Dl.

Preterm birth was defined as delivery prior to 37 weeks of gestation. Low birth weight (LBW) was defined as a birthweight less than 2.5 kg at term. Preterm birthweights were stratified according to gestational age. The WHO definition of abortion was used (loss of pregnancy before 20 weeks) [14].

Perinatal outcomes for women who delivered in the hospital (preterm deliveries, low birthweight and stillbirth as well as early neonatal death) were recorded by chart review. For women who were discharged undelivered or treated as outpatients, phone numbers and names of nearest health facilities where they planned to deliver were also recorded, and outcome follow up was performed until 7 days post-partum. The seven day follow up was chosen as perinatal mortality is defined as the number of stillbirths and the number of neonatal deaths in the first week of life.

### Data analysis

Maternal socio-demographic, maternal and neonatal complications were presented in frequency tables stratified by malaria severity as well as trimester of infection. Univariate analysis for risk factors for adverse maternal and perinatal outcomes was performed and also stratified by malaria severity and trimester. A p-value less than 0.05 was considered statistically significant. Data entry was done in EpiData (Denmark) and analyzed using SPSS 16 software (IBM, Armonk. NY) Additional data analysis was performed with Stata version 15 (Statacorp, College Station, TX).

### Ethical considerations

The study received ethics approval from the Institutional Research Boards of Butare University Teaching Hospital, Kibagabaga District Hospital and Nyamata District Hospital and from the College of Medicine and Health Sciences/University of Rwanda (IRB #212/ CMHS IRB/2016). Written informed consent was obtained from every participant prior to enrollment. The IRB waived guardian consent of minors due to pregnancy.

## Results

The predominance of patients (80.9%) had simple malaria (p<0.001). Most of the patients were from rural areas (85.4%) and there was no difference in the proportion of severe malaria in rural (18.9%) compared to urban areas (20%). Maternal demographics were stratified by malaria severity in Table 1. Only 4.7% of the cohort were 18 or younger (age range 16 to 44) and nine were less than 18. Age and gravidity did not correlate with disease severity. The severity of the disease correlated with the trimester in which the woman experienced her infection. Malaria was more common in the third trimester (72.4%, p<0.001). Severe malaria was also more common in the third trimester. Out of the 85 cases of severe malaria, 12.9% were in the first trimester, 29.4% in the second trimester and 57.6% were in the third trimester (p<0.001) Table 2.

**Table 1:** Demographics of the study population stratified by type of malaria.

		n	Simple Malaria	Severe Malaria	p value
Variables		446	361(80.9%)	85 (19.1%)	0.001
Age (years)	≤18	21 (4.7%)	14 (66.7%)	7 (33.3%)	
	19 to 30	291 (65.2%)	236 (81.1%)	55 (18.9%)	
	>30	134 (30.0%)	111 (82.8%)	23 (17.2%)	NS
Gravidity	1	262 (58.7%)	209 (79.8%)	53 (20.2%)	
	2–3	73 (16.4%)	59 (80.8%)	14 (19.2%)	
	>3	111 (24.8%)	93 (83.8%)	18 (16.2%)	NS
Residence	Rural	381 (85,4%)	309 (81.1%)	72 (18.9%)	NS
	Urban	65 (14.6%)	52 (80%)	13 (20%)	NS

**Table 2:** Severity of malaria stratified by trimester of pregnancy.

	n	First trimester	Second Trimester	Third trimester	p value
	446	52 (11.7%)	71 (15.9%)	323 (72.4%)	<0.0001
Simple malaria	361 (80.9%)	41 (11.4%)	46 (12.7%)	274 (75.9%)	<0.0001
Severe malaria	85 (19.1%)	11 (12.9%)	25 (29.4%)	49 (57.6%)	<0.0001

Maternal clinical manifestations are presented in Table 3. The predominance of patients had fever at the time of diagnosis (94.4%), 11.7% had severe anemia with hemoglobin less than 7g/dl and moderate or severe thrombocytopenia was present in 21% and 9.6%, respectively. Severe CNS complications were noted in 18 patients (4%) with Glasgow Coma Scale ranging from 3 to 8. There were 9 (2%) maternal deaths, 55.6% were in primigravidas, seven were in the 19–30 age group and 2 were greater than 30 years of age. The overall pregnancy outcomes of the entire cohort are presented in Table 4. A total of 80.5% of the women with malaria in pregnancy had a liveborn and a normal term delivery occurred in only 57.6%. The perinatal mortality (defined as stillbirths and early neonatal deaths) was 8.1% with an abortion rate of 13.5%.

**Table 3:** Maternal clinical manifestations (N = 446).

	n (%)
Fever	421 (94.4%)
Fever and headache	119 (26.7%)
Fever, headache and rigors	134 (30.0%)
Fever, headache and gastrointestinal symptoms	157 (35.2%)
Moderate anemia (Hgb 7–10 g/dL)	135 (30.3%)
Severe anemia (Hgb <7 g/dL)	52 (11.7%)
Moderate thrombocytopenia (platelets 50–80 K/mL)	43 (9.6%)
Severe thrombocytopenia (platelets < 50 K/mL)	51 (11.4%)
Hypoglycemia (glucose < 70 mg/dL)	8 (1.8%)
Renal compromise	14 (3.1%)
Respiratory distress	64 (14.3%)
Glasgow Scale < 11/15	32 (7.2%)
Glasgow Scale < 8/15	18 (4.0%)
Maternal death	9 (2.0%)

**Table 4:** Overall pregnancy outcomes with malaria (N = 446).

	n (%)
Abortion	60 (13.5%)
Preterm birth	111 (24.9%)
Low birthweight	45 (10.1%)
Liveborn	359 (80.5%)
Five minute Apgar < 7 in liveborn	44 (12.3%)
Stillbirth	27 (6.1%)
Early Neonatal Death	9 (2.0%)
Normal term delivery	257 (57.6%)

Neonatal outcomes are stratified by trimester of infection in Table 5. With first and second trimester infection, the chance of having a spontaneous loss prior to 20 weeks was high (69.2% and 33.8%, respectively). If infection occurred in the first trimester, only 15.4% of women had a normal term delivery. The chances of having a normal term delivery improved with each trimester (p<0.001), but even with third trimester infection, only 69.7% had a normal term delivery. A high predominance of stillbirths (69.4%) occurred with third trimester infection. The analysis was also performed excluding abortions since women who had early losses could not have later outcomes, When abortions were excluded, half of women with first and second trimester still had a poor outcome.

**Table 5:** Neonatal outcomes stratified by trimester of malaria infection.

	First trimester	Second trimester	Third trimester	p value
N	52 (11.7%)	71 (15.9%)	323 (72.4%)	<0.0001
Abortion	36 (69.2%)	24 (33.8%)	0	NS
Normal term delivery	8 (15.4%)	24 (33.8%)	225 (69.7%)	<0.0001
Preterm delivery	7 (13.5%)	23 (32.4%)	81 (25.1%)	NS
Low birthweight	5 (9.6%)	1 (1.4%)	39 (12.1%)	NS
Stillbirth/early neonatal death	1 (1.9%)	10 (14.1%)	25 (7.7%)	NS
Poor outcome (including abortion)	44/52 (84.6%)	47/71 (66.2%)	98/323 (30.3%)	<0.0001
Poor outcome (excluding abortion)	8/16 (50%)	24/47 (51.1%)	98/323 (30.3%)	<0.0001

Adverse perinatal outcomes were more common in severe than simple malaria Table 6. Rates of preterm delivery were significantly higher with severe malaria (49.4% vs 19.1%, p<0.001). Stillbirth and neonatal death significantly increased in severe malaria (20% vs. 5.3%, p<0.001). The chances of a normal term delivery with severe malaria was 30.6% compared to 64% with simple malaria (p<0.001). Overall poor outcomes were significantly more common with severe malaria whether abortions were included or excluded from the analysis.

**Table 6:** Comparison of neonatal outcomes between severe and simple malaria.

	Simple Malaria	Severe Malaria	p value
n	361	85	
Abortion	47 (13.0%)	13 (15.3%)	NS
Normal term delivery	231 (64.0%)	26 (30.6%)	<0.0001
Preterm delivery	69 (19.1%)	42 (49.4%)	<0.0001
Low birthweight	32 (8.9%)	13 (15.3%)	0.035
Stillbirth/early neonatal death	19 (5.3%)	17 (20.0%)	<0.0001
Poor outcome (including abortion)	130 (36.0%)	59 (69.4%)	<0.0001
Poor outcome (excluding abortion)	83 (26.4%)	46 (63.9%)	<0.0001

## Discussion

According to the WHO, exposure to malaria in pregnancy is highest in West and Central Africa (35% of pregnancies) followed by East and Southern Africa (20%) and accounts for 16% of all low birthweight children [1]. Malaria is the third most common cause of death among women of reproductive age in Africa and is a significant contributor of preterm birth, low birthweight and other pregnancy complications [15–17]. Our study demonstrates that in Rwanda, malaria in pregnancy is associated with poor outcomes in every trimester and that even simple malaria can cause adverse outcome such as abortion, low birth weight and stillbirth as well as maternal morbidity and mortality. Early pregnancy loss (prior to 20 weeks) was seen in 13.5% of the cohort and the perinatal mortality (defined as stillbirths and early neonatal deaths) was 8.1%. As this is on observational study, pregnancy loss rates among women without malaria are not available for comparison. Adverse events will vary in different regions and likely relate to immunity within a region, the burden of malaria as well as underlying risk factors [2]. Regional differences in care as well as access to healthcare can also affect both maternal and neonatal outcomes. Rwanda is unique as its progress towards malaria eradication in the previous decade made the disease in pregnancy less common and it was not previously felt to be a contributor to perinatal mortality [18]. In 2002, an estimate of the malaria prevalence in pregnancy was 13.6% in 6 different health centers [5]. By 2010, estimates were less than 1% of pregnant women [18]. Current rising rates of malaria in Rwanda may be contributing to poor outcomes in reproductive age women who have not previously acquired immunity [5]. Our study did not demonstrate a difference in infection rates between primigravidas and multigravidas that was previously noted in the literature [1–3]. This may because of the changing rates of malaria over the last 2 decades. Teenage pregnancy is uncommon in Rwanda and the population 18 years of age or less is less than 5% of the cohort. The National Rwanda Health Survey in 2015 documented the teenage pregnancy rate to be 7% [5]. Addressing specific outcomes in this subgroup is hindered by the small sample size.

This study is not generalizable to the whole country as the study was performed in 3 hospitals from different regions and with different referral patterns. Rwanda has a hierarchical system of referral with entry into the health system at the health centers, then to the district hospital and finally to the tertiary referral hospitals. Patients with uncomplicated simple malaria may not be referred unless there is a complication thus inflating complication rates of simple malaria seen at the district and referral hospitals sampled. There is no control group of uninfected women to serve as a comparison group of perinatal outcomes. The cohort does represent a diverse population geographically with endemic malaria. The outcomes seen with severe malaria would be representative of what is seen nationally as all pregnant women with severe malaria are transferred to a referral hospital.

Intermittent preventive treatment in pregnancy has been advocated by the WHO, though there is debate on which medication to use [19–22]. There is currently a randomized control trial in Rwanda to examine the role of screening and treatment of asymptomatic pregnant women compared to routine care, which will examine whether perinatal outcomes are improved [23]. When malaria was less common in Rwanda, intermittent preventive treatment was not felt to be clinically appropriate and its use was stopped in 2008 [6, 24]. Many factors have affected malaria rates in sub-Saharan Africa in the last decade and effective control requires dynamic health policies and economic investment which can integrate the necessary changes as well as deliver effective preventive and therapeutic measures.

## Conclusions

The current study demonstrates that poor pregnancy outcomes are seen with malaria in all trimesters and can be stratified by severity of disease. Overall, less than 60% of infected women in this cohort had a normal term delivery, high rates of early pregnancy loss, stillbirth and neonatal mortality were demonstrated, Given the maternal and neonatal complications associated with malaria, close monitoring over the duration of pregnancy is warranted.

## Supporting information

S1 Data (XLSX)
